# Experimental Polarizability Transition Moments of CO_2_ for Excited Vibrational States

**DOI:** 10.3390/molecules29215103

**Published:** 2024-10-29

**Authors:** Carlos Álvarez, Guzmán Tejeda, José M. Fernández

**Affiliations:** Laboratory of Molecular Fluid Dynamics, Instituto de Estructura de la Materia IEM-CSIC, C/Serrano 121, 28006 Madrid, Spain; g.tejeda@csic.es (G.T.); jm.fernandez@csic.es (J.M.F.)

**Keywords:** Raman spectroscopy, CO_2_, high temperature, polarizability transition moments

## Abstract

The Raman spectrum of CO_2_ from room temperature to 1800 K has been measured in a series of experiments. The differential Raman scattering cross-sections for the fundamental bands at 1285.41 cm^−1^ and 1388.18 cm^−1^ have been obtained from reference bands of H_2_ and N_2_ as intensity standards. The Raman cross-sections of CO_2_ hot bands, involving vibrational energy levels up to 5000 cm^−1^, were derived from those of the fundamental bands. The Raman cross-sections obtained this way were reduced to transition moments of the mean molecular polarizability, which make it possible to simulate the Raman spectrum of CO_2_ up to 2000 K. This paves the way for local or remote diagnostics of CO_2_ in hot environments using Raman based techniques.

## 1. Introduction

CO_2_ is a key molecule on Earth. Despite being a minor component of Earth’s atmosphere, CO_2_ is the main driver of its global warming because of its role in unbalancing the Earth’s radiative budget. On the other hand, it is ubiquitous in combustion processes, and it is involved in many biolgical processes like breathing, photosynthesis, and fermentation. All of these processes do affect significantly the local concentration of CO_2_ at the Earth’s crust level, leading, e.g., to day–night or summer–winter oscillations. CO_2_ is also the main component of Venus and Mars atmospheres, and its presence has been detected in hot Jupiter exoplanets [1,2]. Most of these environments are much hotter than Earth, reaching temperatures over 1000 K.

Remote detection of CO_2_ is carried out by IR absorption and emission techniques. Here on Earth, CO_2_ content is continuously monitored from satellites and airborne stations [3]. However, those techniques lack spatial resolution along the absorption path, which is needed for a proper modeling and short-term predictions of CO_2_-affected phenomena at the local scale.

Raman spectroscopy, on the contrary, is able to provide spatial resolution. It has been used to probe CO_2_ in supersonic jets [4,5,6,7], with resolutions of a few microns, and in combustion flames [8,9] with resolutions of millimeters. In turn, Raman LIDAR (LIght Detection And Ranging), a variant of remote sensing, is able to provide spatial resolution below 1 km. Actually, Raman LIDAR is now a leading technology for the atmospheric profiling of water vapor [10,11] and temperature [12,13], and it has been demonstrated for the measurement of CO_2_ [14,15,16]. For the practical detection of CO_2_ using any Raman based technique, the intrinsic intensities of the Raman spectral bands are mandatory. Although the absolute Raman intensities of the two fundamental bands of CO_2_ are known with enough accuracy [17], reliable experimental intensity data for their vibrational hot bands are missing.

There have been several theoretical attempts to simulate the Raman spectrum of CO_2_. The rovibrational Raman intensities of CO_2_ at room temperature were analyzed, taking into account all possible interactions between the rovibrational states [18,19], and the same approach was used to calculate the temperature dependence of the Q-branch intensities of the first hot bands up to 650 K [20]. More recently, an algebraic approach for the vibrational Hamiltonian has been used to simulate the Raman spectrum of CO_2_ at temperatures over 1000 K [21,22].

The molecular quantities responsible for the intensity of the vibrational Raman bands are the transition moments of the polarizability. They are also good tests to benchmark high quality ab initio calculations of polarizability surfaces [23,24,25]. In addition, accurate knowledge of the molecular polarizability surface of CO_2_ is of interest to model its refraction index at high temperature [17], the intermolecular interactions responsible for collision-induced absorption and light scattering [26,27], and the formation of clusters [28].

In this work, we report a set of new transition moments of the mean polarizability of CO_2_ for excited vibrational states aimed to simulate the Raman spectrum of CO_2_ up to 2000 K. This paper is structured as follows: in Section 2, the theoretical background of the Raman spectrum of CO_2_ is reviewed. In Section 3 and Section 4, we present and discuss the results of our work. The experimental setup and measures are described in Section 5, and finally, in Section 6, we present some concluding remarks.

## 2. Theoretical Background

**Figure 1:**
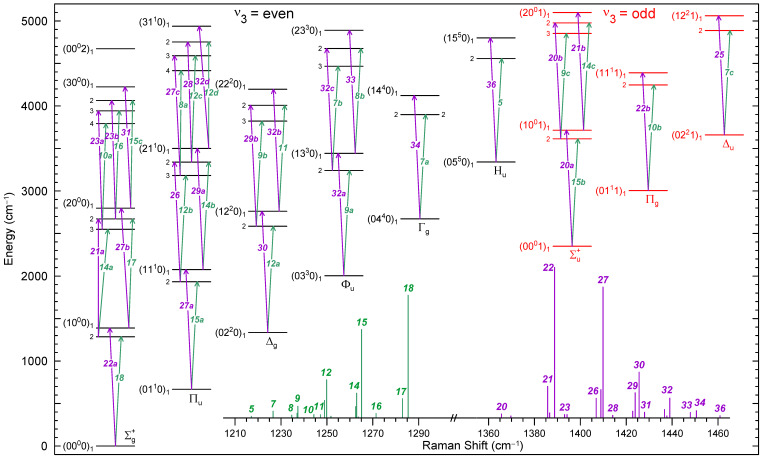
CO_2_ vibrational energy levels diagram up to 5000 cm^−1^ and vibrational Raman transitions observed in this work. Energy levels are grouped vertically by symmetry species in the $D_{\infty h}$ point group. Vertical arrows mark the most intense allowed totally symmetric ($\Sigma_{g}^{+}$) Raman transitions from each vibrational level. Inlet: vibrational Raman bar spectrum at 950 K with the numbering devised from Ref. [8].

CO_2_ is a symmetric linear molecule (point group $D_{\infty h}$), with four vibrational modes, two of them degenerate [29]. The structure of vibrational energy levels up to ∼5000 cm^−1^ is shown in Figure 1, grouped by columns of the same symmetry species. The vibrational energy levels of CO_2_ can be labeled classically in terms of the (harmonic) normal modes $\nu_{1}$ (symmetric stretch), $\nu_{2}$ (doubly degenerate bending), and $\nu_{3}$ (antisymmetric stretch) as $\left(v_{1},v_{2}^{\ell },v_{3}\right)$, where $v_{m}$ is the number of vibrational quanta in the *m*th normal mode, and $\ell =(v_{2},v_{2}-2,...,1/0)$ is the quantum number of the angular momentum of the bending mode. However, the strong Fermi resonance between levels with the same $\left(2v_{1}+v_{2}\right)$ for a given $v_{3}$ leads to a departure from the normal mode description of the excited vibrational states. This resonance, first described by Fermi [30], is caused by the interaction of the close lying harmonic fundamental $\nu_{1}$ and overtone $2\nu_{2}$—through a cubic term $\left(\partial^{3}V/\partial q_{1}\partial q_{2}^{2}\right)$ of the intramolecular potential *V*, where $q_{1}$ and $q_{2}$ are the corresponding nomal coordinates—spliting away the energy levels by ∼100 cm^−1^. This interaction scrambles completely the harmonic vibrational wavefunctions, and it translates in the spectrum as a borrowing of intensity from the fundamental band to the overtone. Furthermore, the strong mixing of the harmonic wavefunctions within the $\left(2v_{1}+v_{2}\right)$ polyads hinders to estimate the transition moments in the harmonic approximation. For that reason, an alternate notation ${\left(v_{1},v_{2}^{\ell },v_{3}\right)}_{r}$ will be used in this work, like in the HITRAN database [31], by labeling each $\left(2v_{1}+v_{2}\right)$ polyad with the harmonic tetrad of its highest component plus a fifth number *r* denoting the individual components of the polyad in decreasing order.

The Raman-allowed transitions due to the totally symmetric $\Sigma_{g}^{+}$ components of the molecular polarizability, the only ones giving rise to prominent Q-branches (ΔJ=0) in the spectrum, would connect levels within each vertical block of the same symmetry. The two most intense Raman transitions departing from each vibrational energy level are depicted as vertical arrows in Figure 1. The corresponding Raman spectrum consists of two separate regions, lower and upper, split by 100 cm^−1^ by the Fermi resonance, as shown in the bar graph inset in Figure 1. The fundamental bands (the Fermi doublet [30]) are those at 1285.41 cm^−1^ and 1388.18 cm^−1^, which are numbered in Figure 1 as 18 and 22, respectively.

The main quantities measured in this work are the intensities of the different Raman lines and bands of CO_2_. The scattered power *I* [W/sr] due to a Raman transition at wavenumber ν over a differential solid angle δΩ [sr] along a direction is given by [32]

(1) $$I\left(\nu \right)=I_{0}\;\;N\;\left(\frac{\partial \sigma (\nu )}{\partial \Omega }\right),$$

where $I_{0}$ is the incident irradiance [W/m^2^], N is the number of molecules in the scattering volume, and (∂σ/∂Ω) is the differential Raman scattering cross-section [m^2^/sr] per molecule. This Raman cross-section depends on instrumental factors like the scattering geometry and the wavenumber $\nu_{0}$ of the exciting radiation, as well as on intrinsic molecular factors like the particular f←i transition, among others.

In particular, for a 90° scattering geometry and Cartesian polarizations M,N={X,Y,Z} of incident (*M*) and scattered (*N*) photons, the differential Raman scattering cross-section for a transition from the initial *i* to the final *f* rovibrational states can be expressed, in S. I. units, as [33]

(2) $${\left(\frac{\partial \sigma }{\partial \Omega }\right)}_{f\leftarrow i}^{MN}={\left(\frac{\pi }{\epsilon_{0}}\right)}^{2}\;{\left(\nu_{0}-\Delta \nu \right)}^{4}\;P_{i}\;L_{f\leftarrow i}^{MN},$$

where $\epsilon_{0}$ is the vacuum permittivity, Δν is the wavenumber of the Stokes–Raman transition, and $P_{i}$ is the temperature-dependent, normalized population of the initial state, which at thermal equilibrium is given by the Boltzmann distribution. The last factor $L_{f\leftarrow i}^{MN}$ in Equation (2) depends solely on molecular properties, and it is called the line strength or scattering coefficient in the literature. More precisely, $L_{f\leftarrow i}^{MN}$ is given by the squared transition moments $\langle f|\alpha_{MN}{|i\rangle }^{2}$ of the 3×3 molecular polarizability tensor α between the initial |i〉 and the final |f〉 molecular rovibrational wavefunctions.

For gaseous samples in the absence of electric or magnetic fields, $L_{f\leftarrow i}^{MN}$ is the result of averaging $\langle f|\alpha_{MN}{|i\rangle }^{2}$ over all molecular orientations times the magnetic degeneracy. Those averages can be expressed as functions of two rotational invariants of the polarizability tensor: the mean $\bar{\alpha }$ and the anisotropy γ. For a linear molecule like CO_2_, these are given by

(3) $$\bar{\alpha }=\left(2\alpha_{xx}+\alpha_{zz}\right)/3$$

(4) $$\gamma =|\alpha_{zz}-\alpha_{xx}|,$$

where *z* is the molecular axis.

Two particular cases are relevant for the present work. First, there are the (J+2)←J Stokes rotational transitions, which can be observed at parallel (M=N) or crossed (M⊥N) polarization. For linear molecules, the Raman cross section is given by

(5) $${\left(\frac{\partial \sigma }{\partial \Omega }\right)}_{(J+2)\leftarrow J}^{MN}={\left(\frac{\pi }{\epsilon_{0}}\right)}^{2}\;{\left(\nu_{0}-\Delta \nu \right)}^{4}\;P_{J}\;\frac{3+\delta_{MN}}{45}\;\frac{3(J+1)(J+2)}{2(2J+1)(2J+3)}\;\langle 0|\gamma {|0\rangle }^{2},$$

where *J* is the rotational quantum number of the initial state and 〈0|γ|0〉 is the polarizability anisotropy of the vibrational ground state v=0. Second, the Q-branches (ΔJ=0) of $v^{\prime }\leftarrow v$ vibrational bands, like those depicted in Figure 1, added up over all *J*s. Their Raman cross-section observed at parallel (M=N) polarization is given by

(6) $${\left(\frac{\partial \sigma }{\partial \Omega }\right)}_{Q(v^{\prime }\leftarrow v)}^{MM}={\left(\frac{\pi }{\epsilon_{0}}\right)}^{2}\;{\left(\nu_{0}-\Delta \nu \right)}^{4}\;P_{v}\;\left(\langle v^{\prime }|\bar{\alpha }{|v\rangle }^{2}+\frac{4}{45}\chi \langle v^{\prime }|\gamma {|v\rangle }^{2}\right),$$

where χ=0.25 is the fraction of anisotropy scattering in the Q-branch for a linear molecule [34]. This expression brings the direct relation of the differential Raman scattering cross-section with the polarizability transition moment $\langle v^{\prime }|\bar{\alpha }|v\rangle$ between the vibrational levels.

It must be remarked that Equations (2), (5) and (6) apply to a definition of the scattering cross section, σ, given by Equation (1), i.e., as scattered power [W] per incident irradiance [W/m^2^]. Currently, Raman scattering is detected as events (counts) proportional to the photon rate. This has caused in the literature an alternate definition of the scattering cross section, $\sigma^{\prime }$, as scattered photon/s per incident photon/(s m^2^). In this latter case, the factor ${\left(\nu_{0}-\Delta \nu \right)}^{4}$ is replaced by $\nu_{0}{\left(\nu_{0}-\Delta \nu \right)}^{3}$. However, the two cross sections, σ and $\sigma^{\prime }$, are different quantities, related by $\sigma =\sigma^{\prime }\left(\nu_{0}-\Delta \nu \right)/\nu_{0}$, and should not be mistaken for one another.

The Raman cross-section in Equation (6) has contributions from both the trace $\bar{\alpha }$ and the anisotropy γ of the polarizability tensor. The two components can be separated through the depolarization ratio

(7) $$\rho =\frac{{\left(\partial \sigma /\partial \Omega \right)}^{MN}}{{\left(\partial \sigma /\partial \Omega \right)}^{MM}}=\frac{3\chi \langle v^{\prime }|\gamma {|v\rangle }^{2}}{45\langle v^{\prime }|\bar{\alpha }{|v\rangle }^{2}+4\chi \langle v^{\prime }|\gamma {|v\rangle }^{2}}\;,$$

which can be determined experimentally by switching the polarization of the observed scattering.

## 3. Results

**Figure 2:**
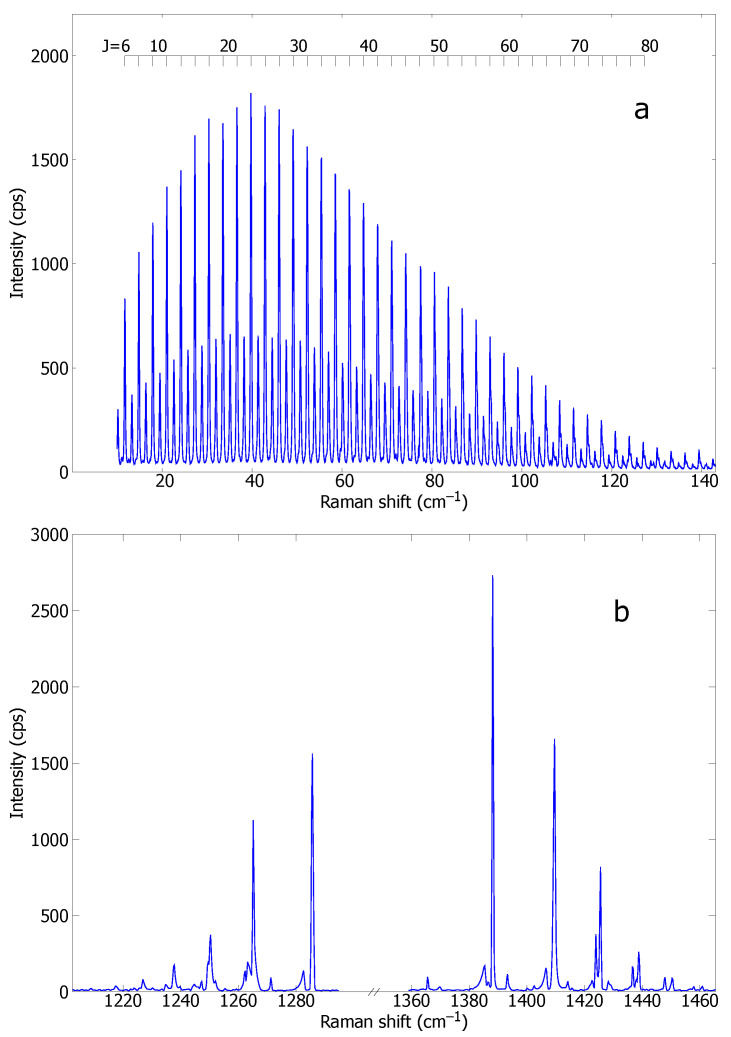
Rotational (**a**) and vibrational (**b**) Raman spectra of flowing CO_2_ against atmospheric air measured at 0.1 mm over the exit of a Ø2.5 iron nozzle heated by an induction coil. The temperature as determined from the rotational spectrum is (950 ± 7) K. The spectral resolutions are 0.4 cm^−1^ (**a**) and 0.7 cm^−1^ (**b**). The relative polarization of incident and scattered photons are (**a**) perpendicular (M⊥N) and (**b**) parallel (MM).

The polarizability transition moments of the fundamental and hot bands of CO_2_ have been obtained by measuring their Raman intensities at known temperatures. In all the experiments, the CO_2_ gas was kept at local thermal equilibrium, and both rotational and vibrational Raman spectra were recorded. Examples of these spectra are shown in Figure 2.

First, the Raman cross-sections of the fundamental bands were measured relative to those of H_2_ and N_2_ intensity standards as

(8) $${\left(\frac{\partial \sigma }{\partial \Omega }\right)}_{{\mathrm{CO}}_{2}}=\frac{S_{{\mathrm{CO}}_{2}}}{S_{\mathrm{ref}}}\frac{F_{\mathrm{ref}}}{F_{{\mathrm{CO}}_{2}}}\frac{p_{\mathrm{ref}}}{p_{{\mathrm{CO}}_{2}}}{\left(\frac{\partial \sigma }{\partial \Omega }\right)}_{\mathrm{ref}}\;,$$

where “ref” stands for the reference gases, *S* is the detected signal, F(ν)=S/I is the spectral sensibility of the instrument, and *p* is the gas pressure. Equation (8) is derived from Equation (1) assuming that the $I_{0}$ and the probed volumes are the same for the two gases. We used the following reference bands of $N_{2}$ and H_2_ as intensity standards:

1. The Q-branch of the fundamental band of N_2_ at 2331 cm^−1^, whose cross-section is ${\left(\partial \sigma /\partial \Omega \right)}_{Q}^{MM}=3.70\times 10^{-35}$ m^2^ ${\mathrm{sr}}^{-1}$ at 532 nm [32,35].
2. The S(1) at 586 cm^−1^ and S(2) at 813 cm^−1^ rotational lines of H_2_, whose cross-sections have been calculated from Equation (5) using the ab initio polarizability anistropy of Ref. [36]. For the MM parallel polarization at 532 nm and 294.4 K, the cross-sections are ${\left(\partial \sigma /\partial \Omega \right)}_{S(1)}^{MM}=6.106\times 10^{-35}$ m^2^ ${\mathrm{sr}}^{-1}$ and ${\left(\partial \sigma /\partial \Omega \right)}_{S(2)}^{MM}=8.93\times 10^{-36}$ m^2^ ${\mathrm{sr}}^{-1}$.

The observed cross-sections for the CO_2_ Q-branches ${\left(\partial \sigma /\partial \Omega \right)}_{Q}^{MM}$ of Equation (6) obtained this way, include a small contribution (∼5%) from the polarizability anisotropy γ. The dominant transition moment $\langle v^{\prime }|\bar{\alpha }|v\rangle$ has been retrieved through Equation (7) for the lower and upper fundamental bands by employing the depolarization ratios of the Q-branches ρ=0.042 and ρ = 0.029 [37], respectively. The obtained values are $5.56\left(5\right)\times 10^{-42}{\mathrm{CV}}^{-1}m^{2}$ and $6.87\left(8\right)\times 10^{-42}{\mathrm{CV}}^{-1}m^{2}$ for the lower and upper fundamental bands of the Fermi doublet, respectively. These values are pretty consistent with the those of the literature [17], $5.58\left(7\right)\times 10^{-42}{\mathrm{CV}}^{-1}m^{2}$ and $6.79\left(9\right)\times 10^{-42}{\mathrm{CV}}^{-1}m^{2}$.

For the hot samples, the local temperature was determined in all cases from the rotational Raman spectrum. The rotational populations $P_{J}$ were obtained from the intensities of the rotational lines through Equation (5), and the $P_{J}$ values were fitted to a Boltzmann distribution of rotational energies. This methodology has proved to be very accurate to ∼1% [7,8].

By measuring the intensities of the different hot bands in each region of the Fermi resonance and comparing them with those of the corresponding fundamental band, the polarizability transition moments were retrieved as

(9) $$\langle v^{\prime }|\bar{\alpha }{|v\rangle }^{2}=\frac{S_{v^{\prime }v}}{S_{f0}}\;\frac{F_{f0}}{F_{v^{\prime }v}}\;\frac{\nu_{f0}^{4}}{\nu_{v^{\prime }v}^{4}}\;\frac{P_{0}}{P_{v}}\;\langle f|\bar{\alpha }{|0\rangle }^{2},$$

where $\nu =\nu_{0}-\Delta \nu$ is the wavenumber of the Raman scattered photon, and the subscript f0 refers to the fundamental bands and $v^{\prime }v$ to the hot bands. The normalized vibrational populations $P_{v}$ were calculated at the local temperature of each sample, as measured from the rotational Raman spectrum.

Raman spectra of CO_2_ have been recorded from samples at 295 K, 373 K, 580 K, 950 K, and 1780 K. The higher the temperature, the larger the number of hot bands in the spectrum. Only 4 bands are observed at room temperature in each region of the Fermi resonance, while at 950 K we were able to measure a total of 15 and 21 bands in the lower and upper regions, respectively. Some $\langle v^{\prime }|\bar{\alpha }|v\rangle$ could be measured at all temperatures, while others only at high or low temperatures. Band overlapping became a limitation at higher temperatures. The values obtained for each $\langle v^{\prime }|\bar{\alpha }|v\rangle$ at the different temperatures were then averaged, and their uncertainty was estimated through the standard deviations.

The 38 transition moments of the mean polarizability of CO_2_ measured in this work are listed in Table 1, where the Raman transitions are arranged according to increasing wavenumber.

## 4. Discussion

The transition moments of Table 1 connect vibrational energy states up to 5000 cm^−1^. Despite having been measured from different experiments at different temperatures, the dispersion is, on average, less than 5%. Many isolated and well-determined bands in the spectrum, like those at 1247.19 cm^−1^ (#11), 1248.99 cm^−1^ (#12b), 1262.50 cm^−1^ (#14b), 1385.73 cm^−1^ (#21a), 1386.62 cm^−1^(#22b), and 1450.55 cm^−1^ (#34), have uncertainties around 3%, despite some of them involving energy levels well above 2000 cm^−1^.

The largest uncertainties correspond to overlapping bands where the contribution of the individual transitions could not be correctly assessed. In some favorable cases, like in the band at ∼1409 cm^−1^ (#27) a bottom-up approach could be applied, where the dominant contribution from #27a at 1409.48 cm^−1^ was determined at room temperature, and its intensity at higher temperature was subtracted from the observed composite band. Therefore, the companion band, #27b at 1408.96 cm^−1^, could not be determined better than 30%. But, other overlapping bands like #23 at 1393 cm^−1^ could not be disentangled, and the intensity was split into halves. The transition moments corresponding to some weak or overlapping bands that could not be determined are listed as blanks in Table 1.

To our knowledge, only one theoretical calculation of the transition moments of the mean polarizability for excited vibrational states of CO_2_ has been published [21]. Such transition moments were calculated from vibrational wavefunctions obtained through an algebraic model in terms of symmetry internal coordinates, combined with the derivatives of $\bar{\alpha }$ with respect to these symmetry coordinates, that were fitted to reproduce the experimental transition moments of Ref. [17]. These theoretical results from the literature are also shown in Table 1 for comparison. In general, the difference between our experimental polarizability transition moments and those from the literature is less than 10%, although this difference tends to increase with the energy of the involved vibrational levels. However, some transitions involving low energy levels like those at 1385.73 cm^−1^ (#21a) and 1409.48 cm^−1^ (#27a) differ more than the estimated uncertainty, which could lead to significant differences in the simulated spectrum.

At this point, it is interesting to appreciate the dramatic effect of the Fermi resonance on the polarizability transition moments of CO_2_. In the double (vibrational and electrical) harmonic approximation, the only non-vanishing transition moments are defined as

(10) $$\langle v_{1}+1|\bar{\alpha }|v_{1}\rangle ≃\sqrt{(v_{1}+1)/2}\;\;\frac{\partial \bar{\alpha }}{\partial q_{1}},$$

where $q_{1}$ is the dimensionless normal coordinate of the symmetric stretch, i.e., the squared transition moments grow linearly with the quantum number $v_{1}$ of the final vibrational state. However, it can be seen in Table 1 that, despite mild growing of both experimental and theoretical polarizability transition moments with the energy, they remained more or less distributed in an unpredictable way. This is the result of the extreme anharmonicity of the vibrational energy levels of CO_2_ due to the Fermi resonance.

**Figure 3:**
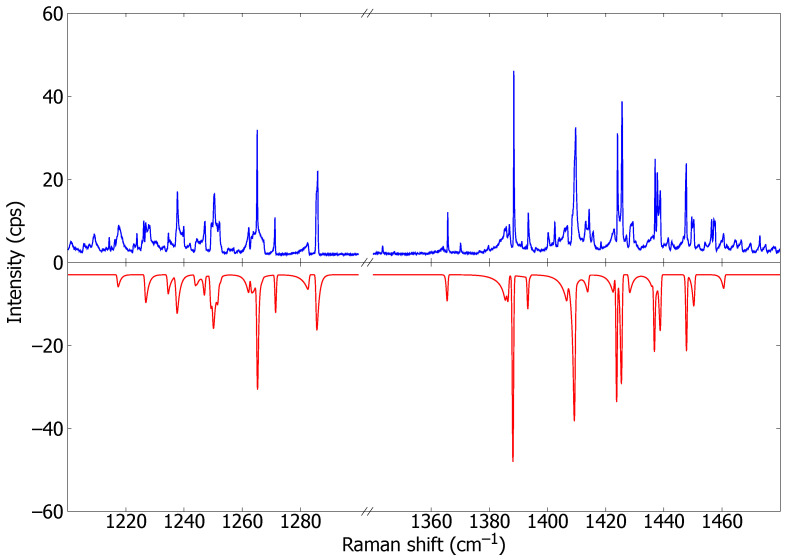
Vibrational Raman spectrum of CO_2_ at 1780 ± 23 K in a CO_2_-enriched CH_4_ + O_2_ flame measured at 1 mm over the tip of the combustion cone. Top: experimental; bottom: simulation. The spectral resolution is 0.7 cm^−1^. The relative polarization of incident and scattered photons is parallel (MM).

The Raman spectrum at 1780 ± 23 K from a CO_2_-enriched CH_4_ + O_2_ flame, shown in the upper panel of Figure 3, has too many overlapping hot bands. Thus, it was not possible to measure many individual bands. We instead used this spectrum to check the validity of our results by comparing it with a simulated spectrum, as shown in the bottom panel of the figure. This latter spectrum was simulated as follows. For each $\left(v^{\prime },J\right)\leftarrow \left(v,J\right)$ transition of the Q-branch, Raman shifts were calculated from the rigid rotor energies of the rovibrational levels

(11) $$E\left(v,J\right)=G_{v}+B_{v}J\left(J+1\right),$$

where the vibrational terms $G_{v}$ and the rotational constants $B_{v}$ were taken from the literature [38]. The intensity of each transition was calculated fom Equation (6), considering only the transition moments of the mean polarizability in Table 1 and weighted by the rotational population $P_{J}$. The stick spectrum obtained that way was convoluted with a Gaussian function of 0.7 cm^−1^ full width at half maximum. However, *J*-dependency of the Fermi resonance, not considered in the simulation, distorts the rigid rotor pattern of the energy levels, and it affects the profile of some of the Q-branches, like that of the fundamental band at 1285.48 cm^−1^ [39].

The simulated spectrum in Figure 3 matches to a great extent the experimental one. Although many new hot bands appear at the temperature of the flame, most of the overlapping bands are properly simulated, like those at 1262 cm^−1^ and 1385 cm^−1^. Most of the differences in band shapes between the experiment and simulation can be attributed to the rigid rotor modeling of the Q-branches, but the integrated intensities are preserved. The new hot bands that appear in the experimental spectrum and not in the simulation originate from vibrational states higher than 3500 cm^−1^, which were not considered in this work.

It is worth noting that the polarizability transition moments of the present work have been determined in experiments at a maximum temperature of 950 K, while the simulation was at 1780 K. Furthermore, the experimental transition moments have been completed with the theoretical ones when the former could not be determined (blanks in Table 1). However, these transition moments correspond to weak bands in the observed spectrum, and their possible inaccuracy seems to not affect significantly the simulation. Despite these limitations, the overall agreement of the simulation in Figure 3 is satisfactory.

The bands at 1369.74 cm^−1^ and 1259.4 cm^−1^ are due to ^13^C^16^O_2_ and ^16^O^12^C^18^O isotopologues, respectively, which were not included in the simulation. Their polarizability transition moments are listed at the end of Table 1, although neither theoretical nor experimental values have been found to compare.

Finally, it should be reminded that the simulation in Figure 3 was carried out with the transition moments of the mean polarizability, not including the contribution from the polarizability anisotropy. For parallel polarization MM, this latter contribution is small enough to be comparable to the uncertainty of the measured intensities. Therefore, for practical purposes, the data in Table 1 can be used along with this expression

(12) $${\left(\frac{\partial \sigma }{\partial \Omega }\right)}_{Q(v^{\prime }\leftarrow v)}^{MM}={\left(\frac{\pi }{\epsilon_{0}}\right)}^{2}\;{\left(\nu_{0}-\Delta \nu \right)}^{4}\;P_{v}\;\langle v^{\prime }|\bar{\alpha }{|v\rangle }^{2},$$

to obtain the absolute differential Raman scattering cross-sections of the Q-branches of fundamental and hot bands of CO_2_ to predict the vibrational Raman spectrum at temperatures up to 1800 K with enough accuracy.

The transition moments of CO_2_ obtained in this work can also be used to retrieve vibrational populations from the intensities of the observed Raman bands, in systems at non-local thermodynamic equilibrium, like, e.g., shock waves, supersonic jets, or reacting flows. Also, it would be interesting to extend this work to other isotopologues of CO_2_ by measuring enriched samples in hot environments. This would contribute to a deeper knowledge of the polarizability surface of CO_2_ and open the possibility to apply Raman-based techniques to other fields, like geological or astrophysical sciences. Finally, we expect that the present results would encourage future theoretical works focused on the polarizability surface of CO_2_ from both ab initio [40] or variational calculations [38].

## 5. Materials and Methods

Four different experiments have been carried out in this work to measure the Raman spectrum of CO_2_ at different temperatures. The first one was aimed to measuring the absolute intensities of the fundamental Raman bands of the Fermi doublet of CO_2_ at room temperature. The other three experiments were devoted to measure the Raman intensities of the vibrational hot bands with respect to those of the fundamental bands by raising the temperature of CO_2_ in order to populate its excited vibrational states. In all these experiments, the CO_2_ gas was kept at local thermal equilibrium.

All the Raman spectra have been recorded with a 1-meter dispersive double monochromator equiped with 2400 grooves/mm gratings and a high-sensitivity CCD detector [41]. The Raman scattering was excited by a Nd:YVO_4_ cw laser, providing up to 10 W at 532 nm, focused onto the samples by means of a f=35 mm lens. The Raman signal was collected at $90^{\circ }$ to the laser beam by using a f=50 mm photographic lens and an achromatic doublet with a total ×10 magnification. In the first experiment the photographic lens was a Leica *f*/1.0, while in the others we utilized a Nikon *f*/1.8. The relative spectral sensitivity of the spectrometer was determined by means of the spectral irradiance of a 5/21 W tungsten lamp, previously calibrated at the Laboratorio de Fotometría y Radiometría of the Instituto de Optica, IO-CSIC [42] in Madrid. The wavenumber scale was calibrated to ±0.1 cm^−1^ by means of Ne emission lines.

The first experiment was aimed to measure the absolute Raman intensities of the fundamental bands of CO_2_ at 1285.48 cm^−1^ and 1388.18 cm^−1^ by comparison with those of reference bands of H_2_ and $N_{2}$ as intensity standards. The gas samples were contained within a ∼100 L aluminum vacuum chamber, along with the laser focusing and photographic lenses [41]. The large volume of the chamber provides a good thermal and mechanical stability during the experiment, while its gas feeding and pumping capabilities allow it to change the gas sample without modifying the optical focuses. The sample pressure was measured with a capacitance manometer (MKS Baratron, Andover, MA, USA) with an accuracy of ±0.01 mbar, and the temperature was monitored with two type K thermocouples close to the probed volume. Each of the three gases (CO_2_, $N_{2}$, and H_2_) were measured independently so that the measured pressure was representative of the gas density. To that goal, the chamber was thoroughly evacuated (∼10^−4^ mbar), flushed with the gas to be measured, and then filled with 40 mbar. The exciting power was 5 W, and the Raman spectra were recorded with a linear polarizer plate to select the polarization of the scattered radiation.

In the second experiment, Raman spectra of CO_2_ from room temperature to 383 K were recorded, employing an 40 × 40 × 20 mm^3^ aluminum cell with optical windows. This cell was heated externally with a 100 Ω resistor and a PID controller with ±1 K accuracy. The cell temperature was measured with two type K thermocouples. The cell was filled with 800 mbar of CO_2_ after being previously degassed two times. The excitation laser power was 2 W.

The third experiment was aimed at recording Raman spectrum of CO_2_ at temperatures up to 1000 K in a dense subsonic laminar flow. To this end, an iron nozzle with a Ø2.5 mm inner channel was heated with an induction coil powered by a homemade 120 W RF generator. A low velocity gas flow was produced by injecting CO_2_ against laboratory atmosphere at a rate of 2.8 × $10^{-5}$ mol/s using a mass flow controller. The temperature was varied from 573 K to 950 K by changing the induction coil power. The Raman spectra were excited with 8 W and measured at ∼0.1 mm from the nozzle tip, where no atmospheric air was present.

The fourth experiment consisted of a CO_2_-enriched CH_4_ + O_2_ flame, reaching ∼1800 K. To this end, a controlled mixture of CH_4_, O_2_, and CO_2_ was injected into a commercial burner [8]. A stable flame was produced with 1.5 × $10^{-4}$ mol/s of CH_4_, 3.0 × $10^{-4}$ mol/s of O_2_ (equivalence ratio ϕ=1.0), and 1.6 × $10^{-4}$ mol/s or 3.3 × $10^{-4}$ mol/s of CO_2_. The Raman spectra were excited with 8 W and measured at 1 mm over the tip of the reaction cone.

## 6. Concluding Remarks

In this work, we have measured the transition moments of the mean polarizability of CO_2_ through a series of experiments designed to reach temperatures up to 1780 K while remaining in local thermal equilibrium. The polarizability transition moments of the two fundamental bands of CO_2_ from the literature were verified, and 34 new polarizability transition moments of hot bands involving energy states up to 5000 cm^−1^ plus two isotopologes were obtained. A comparison with theoretical results from the literature was carried out, pointing out some significant differences. The simulated vibrational spectrum of CO_2_ at 1780 K with the transition moments of the mean polarizability determined at temperatures up to 950 K was compared with the experimental one within the estimated uncertainty. This opens the possibility to employ simulations of the Raman spectrum of CO_2_ at moderate spectral resolution (∼1 cm^−1^) as a diagnostic tool up to 2000 K.

We expect these polarizability transition moments to open the possibility to employ techniques based on Raman spectroscopy to detect and measure CO_2_ in hot environments that are either local, like flames, or remote, like the upper layers of Earth’s atmosphere, the atmosphere of Venus, or other extraterrestrial objects.

A new paper on the simulation of the Raman spectrum of CO_2_ at high temperature was published on line by Lill et al. [43]. The new paper reports a comprehensive simulation approach for the Raman spectrum of CO_2_ based on the rovibrational lines, considering both the trace and the anisotropy invariants of the polarizability. The results of the new paper are complementary of those of the present work.

## Figures and Tables

**Table 1:** Transition moments of the mean polarizability of CO_2_ $\langle v^{\prime }|\bar{\alpha }|v\rangle$ in 10^−42^CV^−1^m^2^. Band # according to Figure 1. Energy levels are labeled as ($v_{1}$, $v_{2}^{\ell }$, $v_{3}$${)}_{r}$, as explained in Section 2. Energies $E_{v}$, $E_{v^{\prime }}$, and Raman shift of the transition, Δν, in cm^−1^.

	Initial		Final			$\langle v^{\prime }|\bar{\alpha }|v\rangle$		
**Band #**	v	$E_{v}$	$v^{\prime }$	$E_{v^{\prime }}$	Δν	**Exp. ^*a*^**	**Error ^*a*^**	**Theory ^*b*^**
5	(0, $5^{5}$, ${0)}_{1}$	3340.53	(1, $5^{5}$, ${0)}_{2}$	4557.60	1217.07	7.29	0.76	4.98
7b	(1, $3^{3}$, ${0)}_{2}$	3240.62	(2, $3^{3}$, ${0)}_{3}$	4467.12	1226.49	—	—	7.05
7a	(0, $4^{4}$, ${0)}_{1}$	2671.72	(1, $4^{4}$, ${0)}_{2}$	3898.31	1226.59	6.77	0.38	5.06
8b	(1, $3^{3}$, ${0)}_{1}$	3442.22	(2, $3^{3}$, ${0)}_{2}$	4676.79	1234.57	—	—	5.57
8a	(2, $1^{1}$, ${0)}_{3}$	3181.46	(3, $1^{1}$, ${0)}_{4}$	4416.15	1234.69	7.24	0.49	8.38
9b	(1, $2^{2}$, ${0)}_{2}$	2585.02	(2, $2^{2}$, ${0)}_{3}$	3822.01	1236.99	6.20	0.83	7.13
9a	(0, $3^{3}$, ${0)}_{1}$	2003.25	(1, $3^{3}$, ${0)}_{2}$	3240.62	1237.37	6.37	0.51	5.16
10b	(0, $1^{1}$, ${1)}_{1}$	3004.01	(1, $1^{1}$, ${1)}_{2}$	4247.71	1243.69	—	—	5.25
10a	(2, $0^{0}$, ${0)}_{3}$	2548.37	(3, $0^{0}$, ${0)}_{4}$	3792.68	1244.31	6.26	0.44	8.14
11	(1, $2^{2}$, ${0)}_{1}$	2760.73	(2, $2^{2}$, ${0)}_{2}$	4007.92	1247.19	5.48	0.10	5.79
12b	(1, $1^{1}$, ${0)}_{2}$	1932.47	(2, $1^{1}$, ${0)}_{3}$	3181.46	1248.99	6.73	0.24	7.18
12a	(0, $2^{2}$, ${0)}_{1}$	1335.13	(1, $2^{2}$, ${0)}_{2}$	2585.02	1249.89	5.67	0.48	5.28
12c	(2, $1^{1}$, ${0)}_{2}$	3339.36	(3, $1^{1}$, ${0)}_{3}$	4591.12	1251.76	6.16	0.42	8.28
12d	(2, $1^{1}$, ${0)}_{1}$	3500.67	(3, $1^{1}$, ${0)}_{2}$	4753.45	1252.78	—	—	6.33
14b	(1, $1^{1}$, ${0)}_{1}$	2076.86	(2, $1^{1}$, ${0)}_{2}$	3339.36	1262.50	5.80	0.10	6.12
14a	(1, $0^{0}$, ${0)}_{2}$	1285.41	(2, $0^{0}$, ${0)}_{3}$	2548.37	1262.96	7.24	0.42	7.07
15b	(0, $0^{0}$, ${1)}_{1}$	2349.14	(1, $0^{0}$, ${1)}_{2}$	3612.84	1263.70	3.26	0.65	5.38
15a	(0, $1^{1}$, ${0)}_{1}$	667.38	(1, $1^{1}$, ${0)}_{2}$	1932.47	1265.09	5.52	0.07	5.42
15c	(2, $0^{0}$, ${0)}_{1}$	2797.14	(3, $0^{0}$, ${0)}_{2}$	4064.28	1267.14	—	—	6.68
16	(2, $0^{0}$, ${0)}_{2}$	2671.14	(3, $0^{0}$, ${0)}_{3}$	3942.54	1271.40	8.96	0.49	8.93
17	(1, $0^{0}$, ${0)}_{1}$	1388.18	(2, $0^{0}$, ${0)}_{2}$	2671.14	1282.96	6.99	0.55	6.73
18	(0, $0^{0}$, ${0)}_{1}$	0.00	(1, $0^{0}$, ${0)}_{2}$	1285.41	1285.41	5.60	0.05	5.60
20a	(0, $0^{0}$, ${1)}_{1}$	2349.14	(1, $0^{0}$, ${1)}_{1}$	3714.78	1365.64	7.41	0.51	7.04
21a	(1, $0^{0}$, ${0)}_{2}$	1285.41	(2, $0^{0}$, ${0)}_{2}$	2671.14	1385.73	8.32	0.29	7.68
22b	(0, $1^{1}$, ${1)}_{1}$	3004.01	(1, $1^{1}$, ${1)}_{1}$	4390.63	1386.62	8.88	0.21	7.16
22a	(0, $0^{0}$, ${0)}_{1}$	0.00	(1, $0^{0}$, ${0)}_{1}$	1388.18	1388.18	6.87	0.08	6.90
23b	(2, $0^{0}$, ${0)}_{2}$	2671.14	(3, $0^{0}$, ${0)}_{2}$	4064.28	1393.13	8.26	0.20	10.07
23a	(2, $0^{0}$, ${0)}_{3}$	2548.37	(3, $0^{0}$, ${0)}_{3}$	3942.54	1394.17	7.52	0.18	7.80
26	(1, $1^{1}$, ${0)}_{2}$	1932.47	(2, $1^{1}$, ${0)}_{2}$	3339.36	1406.89	7.63	0.47	7.53
27b	(1, $0^{0}$, ${0)}_{1}$	1388.18	(2, $0^{0}$, ${0)}_{1}$	2797.14	1408.96	6.30	1.91	9.00
27a	(0, $1^{1}$, ${0)}_{1}$	667.38	(1, $1^{1}$, ${0)}_{1}$	2076.86	1409.48	7.58	0.10	7.07
27c	(2, $1^{1}$, ${0)}_{3}$	3181.46	(3, $1^{1}$, ${0)}_{3}$	4591.12	1409.66	—	—	7.74
28	(2, $1^{1}$, ${0)}_{2}$	3339.36	(3, $1^{1}$, ${0)}_{2}$	4753.45	1414.09	9.04	0.51	10.18
29b	(1, $2^{2}$, ${0)}_{2}$	2585.02	(2, $2^{2}$, ${0)}_{2}$	4007.92	1422.89	8.08	0.45	7.52
29a	(1, $1^{1}$, ${0)}_{1}$	2076.86	(2, $1^{1}$, ${0)}_{1}$	3500.67	1423.81	9.07	0.84	9.57
30	(0, $2^{2}$, ${0)}_{1}$	1335.13	(1, $2^{2}$, ${0)}_{1}$	2760.73	1425.60	7.77	0.51	7.20
31	(2, $0^{0}$, ${0)}_{1}$	2797.14	(3, $0^{0}$, ${0)}_{1}$	4225.10	1427.96	11.26	0.43	10.80
32c	(1, $3^{3}$, ${0)}_{2}$	3240.62	(2, $3^{3}$, ${0)}_{2}$	4676.79	1436.17	—	—	7.53
32b	(1, $2^{2}$, ${0)}_{1}$	2760.73	(2, $2^{2}$, ${0)}_{1}$	4197.36	1436.63	9.27	0.36	9.88
32d	(2, $1^{1}$, ${0)}_{1}$	3500.67	(3, $1^{1}$, ${0)}_{1}$	4938.35	1437.68	7.66	1.29	11.46
32a	(0, $3^{3}$, ${0)}_{1}$	2003.25	(1, $3^{3}$, ${0)}_{1}$	3442.22	1438.97	7.86	0.49	7.30
33	(1, $3^{3}$, ${0)}_{1}$	3442.22	(2, $3^{3}$, ${0)}_{1}$	4890.10	1447.88	12.76	0.34	10.09
34	(0, $4^{4}$, ${0)}_{1}$	2671.72	(1, $4^{4}$, ${0)}_{1}$	4122.27	1450.55	8.34	0.23	7.39
36	(0, 5^5^, 0)_1_	3340.53	(1, 5^5^, 0)_1_	4801.37	1460.84	7.73	0.57	7.46
^13^C^16^O_2_	(0, 0^0^, 0)_1_	0.00	(1, 0^0^, 0)_1_	1369.74	1369.74	7.75	0.12	—
^16^O^12^C^18^O	(0, 0^0^, 0)_2_	0.00	(1, 0^0^, 0)_2_	1259.40	1259.40	5.97	0.11	—

^*a*^ This work. ^*b*^ From Ref. [21].

## Data Availability

The data are contained within the article.
